# Sympathetic neurons secrete retrogradely transported TrkA on extracellular vesicles

**DOI:** 10.1038/s41598-023-30728-3

**Published:** 2023-03-04

**Authors:** Ashley J. Mason, Austin B. Keeler, Farah Kabir, Bettina Winckler, Christopher Deppmann

**Affiliations:** 1 Department of Biology, University of Virginia, Charlottesville, VA USA; 2 Department of Cell Biology, University of Virginia, Charlottesville, VA USA

**Keywords:** Neuroscience, Development of the nervous system, Cell biology, Membrane trafficking, Organelles

## Abstract

Proper wiring of the peripheral nervous system relies on neurotrophic signaling via nerve growth factor (NGF). NGF secreted by target organs (i.e. eye) binds to the TrkA receptor expressed on the distal axons of postganglionic neurons. Upon binding, TrkA is internalized into a signaling endosome and retrogradely trafficked back to the soma and into the dendrites to promote cell survival and postsynaptic maturation, respectively. Much progress has been made in recent years to define the fate of the retrogradely trafficked TrkA signaling endosome, yet it has not been fully characterized. Here we investigate extracellular vesicles (EVs) as a novel route of neurotrophic signaling. Using the mouse superior cervical ganglion (SCG) as a model, we isolate EVs derived from sympathetic cultures and characterize them using immunoblot assays, nanoparticle tracking analysis, and cryo-electron microscopy. Furthermore, using a compartmentalized culture system, we find that TrkA derived from endosomes originating in the distal axon can be detected on EVs secreted from the somatodendritic domain. In addition, inhibition of classic TrkA downstream pathways, specifically in somatodendritic compartments, greatly decreases TrkA packaging into EVs. Our results suggest a novel trafficking route for TrkA: it can travel long distances to the cell body, be packaged into EVs, and be secreted. Secretion of TrkA via EVs appears to be regulated by its own downstream effector cascades, raising intriguing future questions about novel functionalities associated with TrkA^+^ EVs.

## Introduction

The development of the sympathetic peripheral nervous system relies on neurotrophic signaling stemming from the interactions between nerve growth factor (NGF) and its cognate receptor, TrkA. NGF secreted from sympathetic target organs like the salivary gland or eye, binds TrkA present on distal tips of axons and is subsequently internalized into a signaling endosome (SE). This SE is then retrogradely trafficked back to the somatodendritic compartment to promote survival, synapse formation, and several other trophic functions^1–3^. Much effort by our group and several others has gone into determining the fate of the retrogradely transported TrkA^+^ SE. We have shown previously that the activated retrogradely trafficked TrkA^+^ SE can induce association with the cytoskeletal protein, Coronin-1a, which slows degradation of TrkA by evading fusion with the lysosome^4^. The TrkA^+^ SE instead undergoes Coronin-1a dependent recycling to the plasma membrane and subsequent re-internalization^4^ of TrkA, a pathway we have termed “retrograde transcytosis.” Interestingly, it has been shown that the TrkA^+^ SE arrives in the soma as a multivesicular body (MVB)^5^.

Morphologically, MVBs are defined by the presence of intraluminal vesicles (ILVs) that are formed through budding-in events from the endosomal limiting membrane. Functionally, MVBs are mature, late endosomal compartments largely destined for degradation. However, it has become increasingly clear that an alternative fate is for MVBs to fuse with the plasma membrane causing the release of their ILVs as extracellular vesicles (EVs). Although the study of EVs has greatly expanded over the past decade, very little is known about EVs secreted by peripheral neurons and what cargos they contain^6^. EVs, derived from either the plasma membrane or the endosomal system, have been implicated in a variety of functions ranging from cargo transport to intercellular signaling^7–10^. Given our previous findings that retrograde TrkA^+^ SEs can fuse with the somatodendritic plasma membrane^4,11^, taken together with the observations that TrkA^+^ SEs can exist as MVBs, we reason that sympathetic neurons may be able to release EVs containing retrograde cargos like TrkA.

In this work, we investigate EVs as a potential trafficking pathway for TrkA by conducting a comprehensive analysis of their secretion from sympathetic neurons. First, we rigorously show that EVs can indeed be secreted from peripheral cells in accordance with the guidelines set forth by the International Society for Extracellular Vesicles (ISEV) in their position paper “Minimal Information for Studies of Extracellular Vesicles” (MISEV)^12,13^. Next, using microfluidic devices (MFDs) and the neurocircuit tracer, WGA, we show that cargo originating in the distal axon can undergo retrograde transport to the soma where it is packaged and released in EVs, thus establishing EV biogenesis as a possible fate of distal axon derived, long-distance signaling endosomes in peripheral neurons. Finally, we show that TrkA, internalized in distal axons, is secreted on EVs in the somatodendritic compartment. Importantly, this secretion is regulated by TrkA-dependent signaling pathways. This work suggests a potential new mode of trophic signaling that may have an impact in the development, maintenance, and pathogenesis of the nervous system.

## Results

### Sympathetic neurons release extracellular vesicles

**Figure 1:**
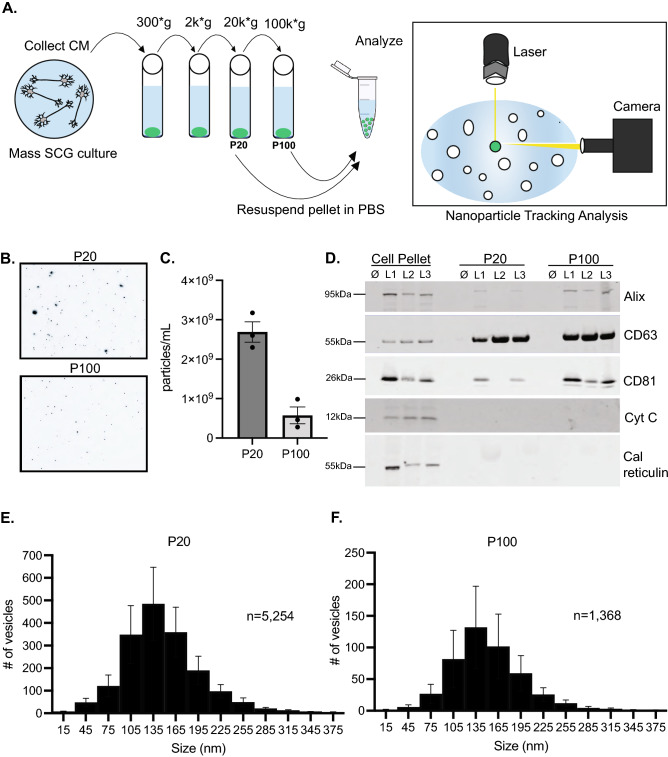
EV isolation and analysis by immunoblot and NTA. (**A**) Schematic of EV isolation from SCG cultures via centrifugation and downstream NTA by ZetaView. (**B**) Still frames captured from NTA videos at t = 30 secs. (**C**) Quantification of the video analysis shown in (**B**). Shown is mean ± SEM for three biological replicates. (**D**) Immunoblot analysis of the canonical EV markers: Alix, CD63, CD81, and the intracellular markers: cytochrome C (mitochondria) and calreticulin (ER). Cell pellet, P20, and P100 fractions from three independent litters (L1, L2, L3) and a “no cell” media control (∅) are shown. These blots are cropped. Distinct blots are demarcated from each other by white spaces. (**E**), (**F**) Size distribution histogram of 5,254 particles from the P20 fraction and 1,368 particles from the P100 fraction for three biological replicates.

We first wanted to determine if EVs are secreted by mouse sympathetic cell cultures. To investigate this, mixed sympathetic cultures containing neurons, satellite glia, and fibroblasts were established for 7 days in vitro (DIV) and EVs were isolated from the conditioned media (CM) using differential centrifugation (Fig. 1A)^13^. We collected two EV fractions: the pellet from the 20,000 × g spin (P20) and the pellet from the 100,000 × g spin (P100) (Fig. 1A). We initially chose to analyze both the P20 and P100 fractions. It is noteworthy that while these fractions sediment vesicles and nanoparticles based on density, they still exhibit significant heterogeneity in terms of EV size and biogenic origin. Both the P20 and P100 fractions were resuspended in PBS for subsequent nanoparticle tracking analysis (NTA) (Fig. 1A,B). Quantification of NTA-tracked particles showed a greater concentration of particles in the P20 fraction compared to the P100 fraction (Fig. 1C). Size distribution histograms from NTA show a mean diameter of 134 nm and 136 nm for the P20 and P100 fractions, respectively (Fig. 1 E,F). To support the notion that these fractions are enriched for EVs, we blotted against canonical EV markers: CD63, CD81, and Alix (Fig. 1D, Supplementary Fig. S1). All three EV-associated markers were detected in the cell pellet, P20 and P100 fractions of three independent mouse litters (litter L1-L3; Fig. 1D). Importantly, cytochrome C, a mitochondrial marker, and calreticulin, an ER resident protein, were not detected in the P20 and P100 fractions, indicating undetectable contamination by intracellular organelles (Fig. 1D, Supplementary Fig. S1). Lastly, neither CD63, CD81, Alix, calreticulin, nor cytochrome C was detected in a “media only” condition (lane: ∅) where no cells were plated (Fig. 1D, Supplementary Fig. S1). We also used NTA to determine the concentration of particles in this “media-only” condition where no cells were plated. We observed 5.23 × 10^7^ ± 5.21 × 10^6^ particles/mL for the P20 fraction and 5.38 × 10^7^ ± 5.07 × 10^6^ particles/mL for the P100 fraction representing 1.94% and 9.36% of the particles observed in Fig. 1C, respectively (Supplementary Fig. S2). Lastly, we conducted a series of solution controls and found that the concentration of particles derived from these sources is minimal (ranging from 1.16 × 10^6^ to 5.50 × 10^6^ particles/mL) (Supplementary Fig. S2). Additionally, we determined the optimal cell density and growth duration necessary to robustly produce and detect EVs in our cultures. We found that we could reliably detect EV markers by immunoblot (Supplementary Fig. S2) and greater than 3 × 10^9^ particles/mL by NTA at starting cell densities above 80,000 cells that were grown for 7 DIV (Supplementary Fig. S2). Therefore, we used greater than 80,000 cells for all experiments.

**Figure 2:**
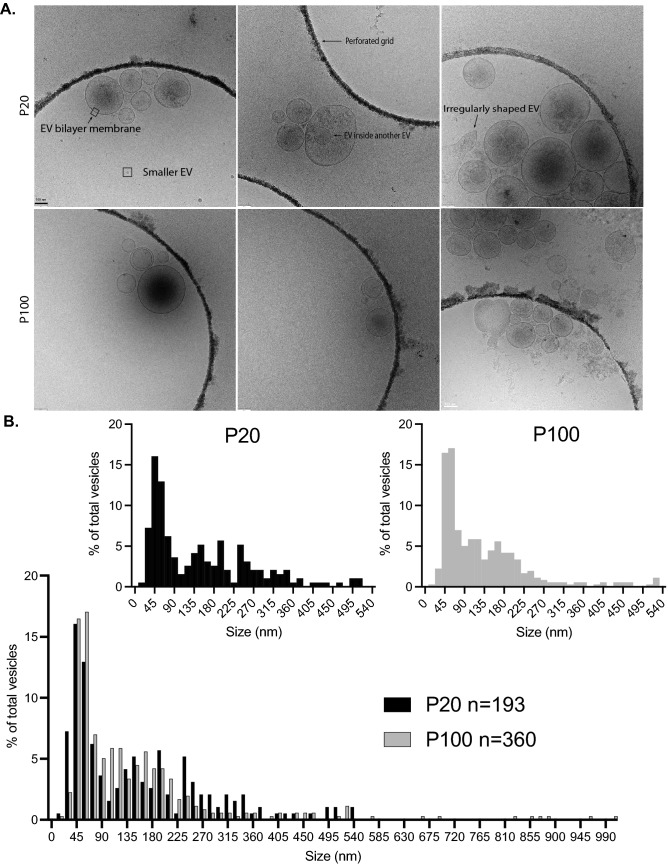
Morphology and sizing of EVs by cryo-EM. (**A**) Cryo-EM micrographs from the P20 (top row) and P100 (bottom row) fractions. Different types of EVs are annotated for easier appreciation of diverse EV morphologies. (**B**) Size distribution histogram for all measured EVs from cryo-EM micrographs (P20: n = 193 EVs, mean diameter 146.62 nm; P100: n = 360 EVs, mean diameter 152.59 nm). The left histogram (black bars) is the sizing of the P20 fraction, and the right histogram (gray bars) is the sizing of the P100 fraction separated out from the histogram below. Data is from three biological replicates and the scale bar is 100 nm for all images.

NTA and immunoblot are good indicators for the presence of EVs in both the P20 and P100 fractions derived from sympathetic cultures, but we wanted to also visualize these isolated EVs by microscopy. Thus, we assessed the size and morphology of sympathetic culture-derived EVs using cryo-transmission electron microscopy (Cryo-EM). We collected high-magnification micrographs of the P20 and P100 fractions and observed many particles delimited by a membrane bilayer, consistent with them being EVs (Fig. 2A). The full-size distribution histogram of both the P20 (black bars) and P100 (gray bars) fractions are shown separately and together (Fig. 2B). For the P20 and P100 fractions the mean EV diameter was 146 nm and 153 nm, and the median EV diameter was 115 nm and 95 nm, respectively. We did not observe any vesicles in micrographs from either the P20 or P100 fractions of “media only” controls where no cells were plated. However, in low magnification micrographs of both the P20 and P100 fractions, we found that the P20 fractions contained large electron-dense aggregates that were difficult to measure as discrete vesicles and were therefore not included in the size distribution histograms (shown in Fig. 2B) (Supplementary Fig. S3). Since the P20 fraction contained these large aggregates, we restricted our analysis to P100 fractions going forward. Lastly, to better contextualize our EVs within the literature, we performed a meta-analysis of the sizes of ILVs and EVs from PC12 cells and sympathetic neurons from published micrographs^5,14^. The sizes of these published EVs and ILVs range from 30 to 110 nm (Supplementary Fig. S4). Furthermore, we conducted a detailed characterization of all particles in our fractions, including those with a single membrane, as well as EVs encapsulated within other EVs (Supplementary Fig. S4).

### Sympathetic EVs contain cargo derived from the distal axon

**Figure 3:**
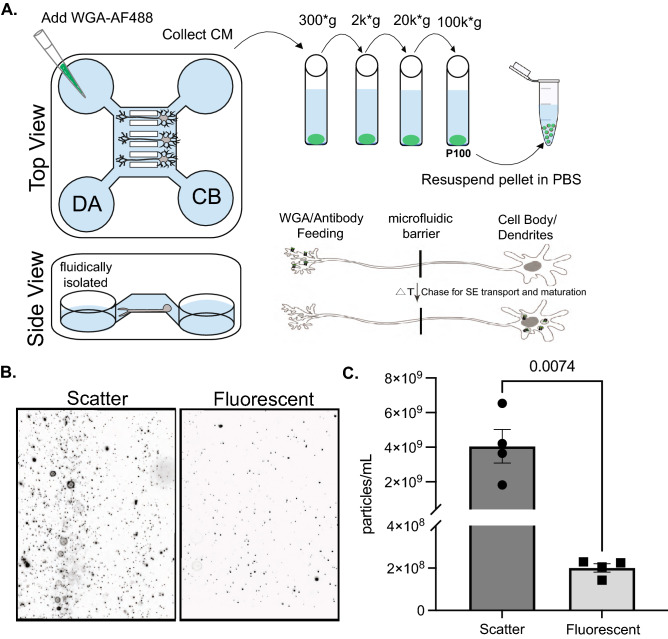
Somatodendritcally secreted sympathetic EVs carry cargo originating in the distal axon. (**A**) Schematic of the WGA-AF488 feeding assay in MFDs. WGA-AF488 was added to the distal axon (DA) chamber which was fluidically isolated from the cell body (CB) chamber. (**B**) Still frames captured from NTA videos at t = 30 secs in scatter and fluorescent mode. (**C**) Quantification of the total number of particles (scatter) and the number of fluorescent (WGA-AF488^+^) particles collected from the P100 fraction after WGA-AF488 addition to the DA chamber of MFDs containing wild-type SCG neurons. Shown is the mean ± SEM for four biological replicates. P values are indicated above the pairwise brackets.

Given that a subset of EVs are derived from an endosomal origin and peripheral neurons can contain axons that extend many microns out from the cell body. We wondered whether cargos that originate in these distal axons can be trafficked back to the cell body and secreted as EVs. To do this, we cultured SCG neurons in microfluidic devices (MFDs) which allowed us to separate the cell bodies (CB) of neurons from their distal axons (DA) by a series of microgrooves (Fig. 3A). Next, under fluidic isolation, we added an AlexaFluor 488 conjugated wheat germ agglutinin (WGA-AF488), a well-known neuronal tracer, to the DA chamber to label SCG neurons at their distal axons (Fig. 3A). We collected and pooled CM from the CB chamber of six MFDs and isolated EVs by differential centrifugation 15 h after adding WGA-AF488 (Fig. 3A). The ZetaView NTA instrument is equipped with a filter allowing measurement of fluorescently labeled particles. We employed this to measure the total number of particles secreted (scatter) from the sympathetic cultures and the number of WGA-AF488^+^ particles secreted from SCG neurons (fluorescent) (Fig. 3B,C). By scatter, 4.05 × 10^9^ ± 9.71 × 10^8^ particles/mL were detected with 2.01 × 10^8^ ± 1.99 × 10^7^ WGA-AF488^+^ particles/mL (Fig. 3C). Importantly, both scatter and fluorescent particle counts from a media-only condition where no cells were plated in MFDs were minimal, indicating that WGA-AF488 does not diffuse against the microfluidic isolation from the DA to the CB chamber in the absence of cells (Supplementary Fig. S5). Based on these findings we conclude that cargo originating in the distal axon can retrogradely traffic in the axon and be released as EVs from the somatodendritic domain.

### Sympathetic EVs contain active retrogradely trafficked TrkA receptors

**Figure 4:**
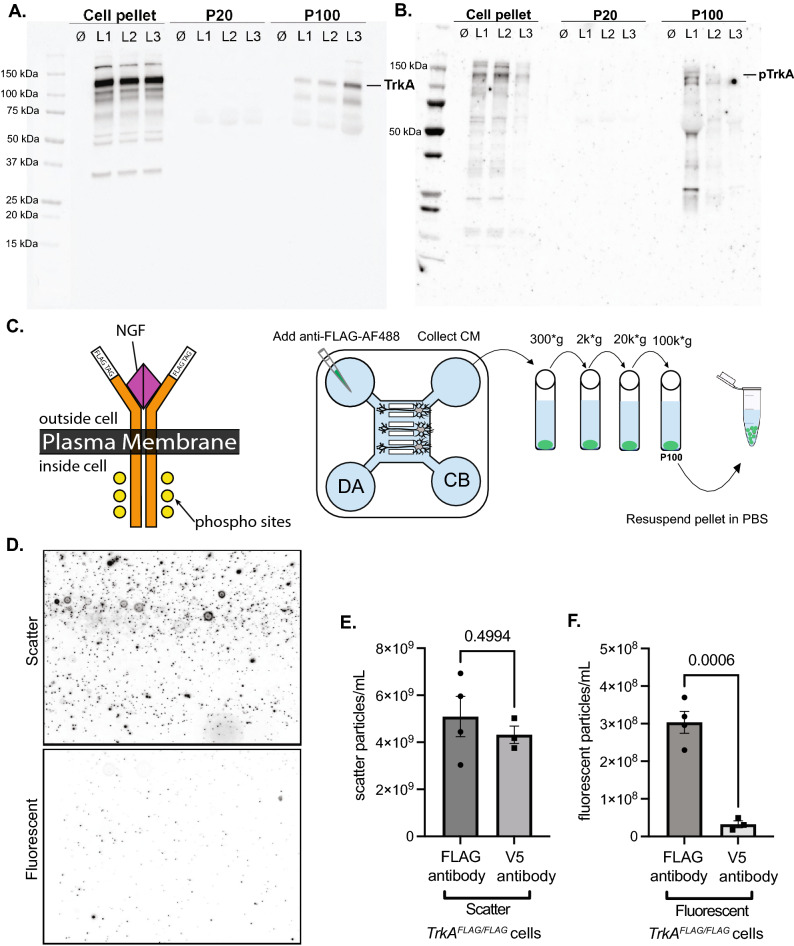
Somatodendritically secreted sympathetic EVs contain the TrkA receptor that originated in the distal axon and was transported retrogradely to the soma. (**A**)**, **(**B**) Full immunoblot analysis of TrkA and phosphorylated TrkA (Y-490) in cell pellets, P20 and P100 EV fractions from three independent litters (L1, L2, L3) and a “no cell” media control (∅). *Note*: the three litters from (**A**) are different from the three litters from (**B**). (**C**) Schematic showing the topology of FLAG-TrkA expressed from the mouse knockin locus in *TrkA*^*FLAG/FLAG*^ mice (NGF: magenta triangle, phosphorylation sites: yellow circles). Sympathetic neurons from *TrkA*^*FLAG/FLAG*^ mice were grown in MFDs. Anti-FLAG-AF488 antibody was fed to the distal axons (DA) and 15 h later CM was collected from the cell body (CB) compartment and EVs were isolated by differential centrifugation. (**D**) Still frames captured from NTA videos at t = 30 secs in scatter (top) and fluorescent (Anti-FLAG-AF488^+^) (bottom) mode. (**E**)**, **(**F**) Quantification of the number of scatter or fluorescent (AF488 +) particles detected after either Anti-FLAG-AF488 or V5-AF488 addition to the DA chamber of MFDs containing *TrkA*^*FLAG/FLAG*^ SCG neurons. Shown is the mean ± SEM for four biological replicates. P values are indicated above the pairwise brackets.

Once we determined that axonally derived cargos could be secreted in EVs, we wanted to determine if TrkA, a well-established retrogradely trafficked endosomal cargo, could be recovered in EVs from sympathetic neurons. Using immunoblotting of P20 and P100 EVs derived from SCG cells grown in mass culture (three separate litters L1–L3), we detect TrkA (Fig. 4A) on purified EVs in the P100 fraction. Next, we wanted to determine if the TrkA that was secreted on EVs was phosphorylated and therefore activated. We repeated the experiment with three additional litters and observed that pTrkA was detectable in the P100 EV fraction, indicating that some activated TrkA is secreted on EVs (Fig. 4B).

In order to determine whether axonally derived TrkA could be packaged into EVs upon retrograde arrival to the soma, we employed the MFD setup as described in Fig. 3A. We made use of a mouse line containing a FLAG tag that was knocked in frame with the extracellular domain of the TrkA locus (*TrkA*^*FLAG/FLAG*^ transgenic mice) (Fig. 4C)^15^. This allows selective labeling of pre-endocytic TrkA receptors on the distal axon plasma membrane by adding an anti-FLAG antibody conjugated to AlexaFluor 488 (anti-FLAG-AF488) just to the DA chamber of the MFD as previously reported (Fig. 4C)^16^. Fifteen hours after anti-FLAG-AF488 antibody addition to the DA, we collected and pooled the CM from the CB chamber of six MFDs to isolate EVs (Fig. 4C). Using scatter and fluorescent NTA, we detected 5.09 × 10^9^ ± 8.56 × 10^8^ total particles/mL and 3.03 × 10^8^ ± 2.91 × 10^7^ fluorescent particles/mL after the addition of anti-FLAG-AF488 antibody (Fig. 4D–F). To ensure that these fluorescent particles were indeed anti-FLAG antibodies bound to TrkA and not due to non-specific uptake of anti-FLAG antibody, we added an irrelevant anti-epitope antibody, anti-V5-AF488, to *TrkA*^*FLAG/FLAG*^ cells (Fig. 4E,F). As expected, the anti-V5-AF488 antibody did not significantly change the total number of particles secreted (4.32 × 10^9^ ± 3.71 × 10^8^ particles/mL, Fig. 4E) nor did it result in significant fluorescent particle detection (3.24 × 10^7^, ± 9.16 × 10^6^ particles/mL, Fig. 4F) indicating that the anti-V5-AF488 antibody was not non-specifically endocytosed at significant levels. Next, we added an anti-FLAG-AF488 antibody to wild-type sympathetic cultures which do not express the *TrkA*^*FLAG/FLAG*^ gene. We did not detect significant fluorescent particles thus confirming that the anti-FLAG-AF488 needed to bind the FLAG epitope on the FLAG-tagged TrkA receptor to be internalized and secreted as EVs (Supplementary Fig. S5).

### Inhibition of phosphoinositide 3-kinase and phospholipase-C-γ reduces the number of TrkA EVs

**Figure 5:**
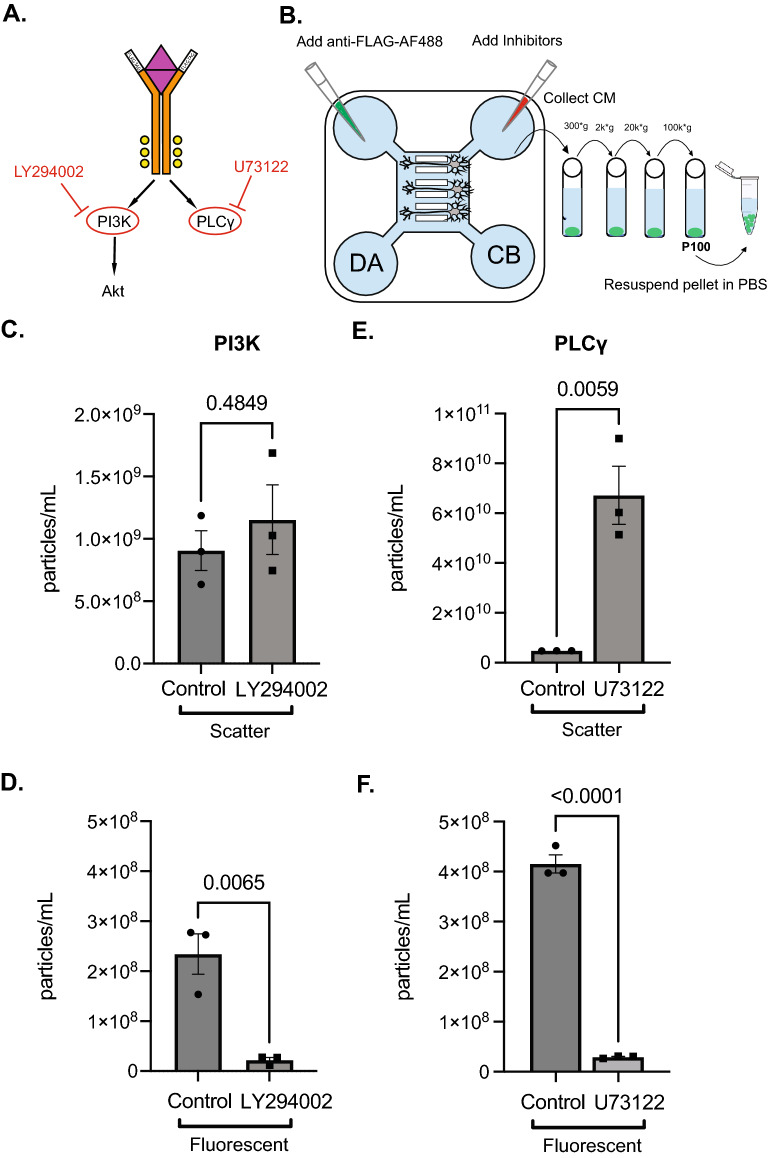
Inhibition of TrkA-dependent signaling pathways suppresses TrkA^+^ EV release. (**A**) Schematic of the downstream pathways activated by TrkA phosphorylation and inhibitors that block them. (**B**) Schematic of inhibitor and Anti-FLAG-AF488 antibody application to compartmentalized neurons to generate TrkA^+^ EVs. (**C**)**, **(**D**) Quantification of the total number of scatter or fluorescent (Anti-FLAG-AF488^+^) particles detected after anti-FLAG-AF488 antibody addition to the DA chamber of MFDs containing *TrkA*^*FLAG/FLAG*^ SCG neurons treated with the PI3K inhibitor, LY294002. (**E**)**, (F**) Quantification of the total number of scatter or fluorescent (Anti-FLAG-AF488^+^) particles detected after anti-FLAG-AF488 antibody addition to the DA chamber of MFDs containing *TrkA*^*FLAG/FLAG*^ SCG neurons treated with the PLC-ɣ inhibitor, U73122. Shown is the mean ± SEM for three biological replicates. P values are indicated above the pairwise brackets.

The ligand-bound activated TrkA receptor transduces its signal via several canonical downstream pathways such as phosphatidylinositol-3-kinase (PI3K) and phospholipase-C-ɣ (PLC-ɣ) (Fig. 5A). Interestingly, these downstream pathways often determine the maturation and trafficking of the TrkA^+^ SE^17,18^. We tested whether these pathways are important for TrkA^+^ EV release. Using *TrkA*^*FLAG/FLAG*^ SCG neurons cultured in MFDs, we simultaneously added anti-FLAG-AF488 to the DA chamber and different inhibitors to the CB chamber for 15 h and then prepared EVs by differential centrifugation from CM collected and pooled from the CB chamber of six MFDs (Fig. 5B). Inhibition of PI3K using the chemical inhibitor, LY294002, did not change the total number of particles secreted (Fig. 5C), but did cause a significant decrease in the number of fluorescent TrkA particles detected in the P100 fraction (Fig. 5D) compared to a DMSO control. Interestingly, inhibition of PLC-ɣ using U73122 caused a significant increase in the total number of particles detected (Fig. 5E). Despite this increase in total particles detected in U73122-treated cells, there was a significant decrease in the number of fluorescent TrkA particles detected compared to control (Fig. 5F). In order to determine if the changes in EV secretion might be due to the inhibitor treatments causing increased cell death and/or morphological and functional alterations, we characterized several indicators of poor health and morphology in DMSO-treated and inhibitor-treated cultures. Etoposide was used to induce cell death as a positive control for our chosen readouts (Supplementary Fig. S6). Importantly, size analysis of the cell bodies of SCG neurons after the different inhibitor treatments did not show any significant differences compared to a DMSO control nor was there any cell loss or evidence of pyknotic nuclei that might account for the differences in EV secretion (Supplementary Figs. S6, S7). In addition, the total cell number did not change between the inhibitor-treated cultures and the DMSO-treated cultures, and neither did the cell counts of SCG neurons, satellite glia cells, and other cells (Supplementary Figs. S6, S7). Lastly, the same number of MAP2 + neurons had internalized and transported anti-FLAG-AF488 antibody from the DA to the CB chamber, indicating that neuron health was not affected by the inhibitor treatment (Supplementary Fig. S7).

## Discussion

Retrograde trafficking of TrkA is essential for sympathetic neuron survival^19,20^. The TrkA^+^ SE has recently been shown to arrive in the cell body from the distal axon as a multivesicular body; the precursor organelle for exosome formation^5^. Furthermore, these TrkA^+^ MVBs were shown to contain activated TrkA receptors that remain in complex with PLC-ɣ^5^. Previously, we have shown that the retrogradely trafficked TrkA^+^ SE can undergo recycling to the plasma membrane^4^. In sympathetic neurons, another neurotrophin receptor, p75NTR, has been shown to be routed away from the lysosome and secreted in EVs^14^. Based on these data, we speculated that activated TrkA receptors could be packaged and secreted in EVs. Indeed, using compartmentalized cultures and *TrkA*^*FLAG/FLAG*^ transgenic mice, we show that TrkA originating in the distal axon is retrogradely trafficked to the cell body in SEs and then subsequently secreted in EVs. We thoroughly characterize these sympathetic EVs using nanoparticle tracking analysis, immunoblot assays, and cryo-EM in accordance with MISEV guidelines. Lastly, we demonstrate that activation of different downstream pathways of TrkA affects its secretion in EVs.

### Rigorous application of MISEV guidelines demonstrates that EVs are secreted from sympathetic cultures.

We find through immunoblot, NTA, and cryo-EM analysis that both the P20 and P100 fractions contain a diverse and heterogeneous set of EVs with a range of sizes and densities. We use several controls in all experiments to ensure that we could robustly detect EVs above baseline. In addition to solution controls, we experimented with both cell density and duration in culture. Increasing the density of seeded cells increases EV concentration; however not entirely linearly. We speculate that this is because we do not use any inhibitor of cell division in these sympathetic cultures. As such, these mouse SCG cultures contain a mixture of different cell types such as satellite glia, other glia cells, as well as some fibroblasts and endothelial cells, all of which can secrete EVs. After 7 DIV, the number of mitotic (non-neuronal) cells has increased, likely accounting for the increase in the concentration of EVs at higher cell densities. At 2 DIV the cultures have yet to stabilize and the conditioned media from these samples contains cell fragments and debris from the trituration and cell plating process. These experiments highlight the importance of consistency in all parameters related to EV collection (DIV, density, duration of media conditioning) to accurately compare EV secretion across different conditions or genotypes. These parameters have been thoroughly described within the MISEV guidelines^12^.

### Sympathetic cultures secrete a diverse set of EVs with many sizes.

Size analysis of sympathetic EVs by NTA and cryo-EM shows that the majority of EVs fall below 300 nm in diameter. We use two methods of sizing to measure these sympathetic EVs as both methods have their limitations. The ZetaView NTA measures the hydrodynamic radius of nanoparticles with a resolution limit of around 70–90 nm. Therefore NTA sizing analysis excludes smaller vesicles^21,22^. In contrast, cryo-EM detects smaller EVs, but due to aggregation and concentration issues, larger EVs are excluded from the analysis. Our cryo-EM sizing shows two distinct peaks for both the P20 and P100 fraction, a sharper taller peak centered around 45 nm and a broader, wider peak around 180 nm (Fig. 2B). We measured the size of published sympathetic EVs and found that EVs derived from NGF-differentiated PC12 cells and primary sympathetic cultures were below 100 nm in diameter^14^. Although we were able to measure EVs from other sympathetic neurons, they are not directly comparable to our results because we used Cryo-EM while the published literature used transmission electron microscopy (TEM)^5,14,23^. Lastly, we note that we see small non-lipid bilayer encapsulated EVs by cryo-EM. The EV field is increasingly reporting these small EVs that lack a bilayer as exomeres or extracellular particles that co-isolate with EVs^24–26^. We also see EVs enveloped inside other EVs. There is speculation as to whether these EVs are naturally encapsulated inside each other or whether this is an artifact of ultracentrifugation resulting in membranes fusing into other membranes^23,27^. However, this does not appear to be EVs imaged on a different z-plane from each other since their membranes curve or deform around other EVs (Supplementary Fig. S4).

### Cargos transported retrogradely from the axon can be secreted in EVs from the somatodendritic domain

The origin and packaging of EV cargos in neurons is still relatively unknown^9^. Endosomes are one of the intracellular compartments for trafficking EV cargos within the cell prior to secretion. Due to their high motility, endosomes can traffic cargo both locally and across long distances within neurons^28^. We used a fluorescently labeled lectin, wheat germ agglutinin (WGA), to visualize its trafficking through the cell from the distal axon to the cell body^29^. We show that WGA that originated in the distal axon can be packaged into EVs released at the cell body. WGA is primarily used as a neuronal circuit tracer to trace interconnected neurons;^30^ however, the mechanism by which WGA traverses synapses is still debated^31^. We speculate that these neuronal-derived EVs might serve as one mechanism by which WGA can “hop” the synapse from one neuron to another. Our sympathetic neuronal cultures contain a mixture of all superior cervical ganglion cell types including satellite glia cells which can also secrete EVs. We show that a small proportion of all EVs secreted from our sympathetic cultures contain WGA and therefore are of neuronal origin. Since we only added WGA to the DA of MFDs it is only being internalized by axons of neurons that grew through the microgrooves to reach the DA chamber. We note that we cannot determine what proportion of neuronally secreted EVs contain WGA since the CB chamber from which conditioned media is collected contains many neurons whose axons did not traverse the microgrooves and were thus not able to pick up WGA. Future experimentation will be needed to more precisely determine the proportion of somatodendritically secreted EVs that contain retrograde cargo transported from the axon.

### A novel EV cargo, TrkA, is derived from axonal retrograde endosomes and remains phosphorylated in the secreted EV

We identified TrkA as a neuronally secreted EV cargo in sympathetic cultures by Western blots. Remarkably, TrkA only partitions into the P100 fraction. While a potential function for TrkA^+^ EVs is not currently known, a proportion of these TrkA-containing EVs remains catalytically active as evidenced through phosphorylated TrkA immunoblots, and might thus be capable of signaling in recipient cells. We also showed that TrkA which was internalized in the distal axon was able to be included in EVs secreted from the somatodendritic domain. Since only sympathetic neurons express TrkA and extend axons to the distal axon chamber, the TrkA^+^ EVs are definitely secreted by the sympathetic neurons themselves. The other cell types found in our cultures do not express the TrkA receptor nor do they span the microgrooves of MFDs and do not contribute to TrkA^+^ EV counts in our experiments. We thus discovered a new trafficking route for retrogradely derived TrkA, i.e. secretion in EVs.

### TrkA-positive EV secretion is regulated by signaling cascades in the somatodendritic domain.

The binding of NGF to TrkA triggers autophosphorylation of tyrosine residues located on its intracellular domain thereby initiating a kinase cascade on the downstream effector signaling pathways: Ras/MAPK, PI3K and PLC-ɣ^19,32–35^. Activation of different downstream pathways of TrkA has been shown to affect the trafficking and functional output of the TrkA^+^ SE. For example, blocking PI3K in the distal axon prevents the initiation of retrograde transport of the TrkA^+^ SE^18^. Additionally, recruitment of PLC-ɣ to the TrkA receptor promotes internalization of the ligand-receptor complex in the distal axon^17^. Based on these data, we wanted to test whether TrkA signaling affects the production of TrkA^+^ EVs. Our findings suggest that inhibition of PI3K signaling in the cell body of sympathetic neurons influences the production of TrkA^+^ EVs without affecting total EV secretion. Additionally, inhibition of PLC-ɣ signaling in the cell body of sympathetic neurons reduces TrkA^+^ EVs, but also causes a striking increase in the total number of EVs released. The mechanisms underlying these inhibitors’ effects on EV secretion remain unknown and it is important to consider that PI3K and PLC-ɣ are downstream of several different receptors in addition to TrkA. Lastly, broadly inhibiting these pathways might alter the trafficking and release of EVs independent of the cargo as shown by the increase in the number of particles released when PLC-ɣ is inhibited (Fig. 5E).

In summary, we have rigorously characterized EVs derived from primary sympathetic cultures through protein analysis, cryo-transmission electron microscopy, and nanoparticle tracking analysis. We have shown that EVs released from sympathetic cultures are heterogenous in size and morphology. Our findings thus expand the sparse literature on sympathetic EVs. Finally, we demonstrate the successful isolation of labeled EVs from specific neuronal domains. Specifically, we demonstrate that TrkA internalized at the distal axon can be secreted in EVs from the somatodendritic domain. Future studies investigating the mechanisms underlying TrkA partitioning into secretion-competent organelles will help elucidate the effects different inhibitors have on TrkA^+^ EV numbers. Our results thus demonstrate a novel trafficking route for TrkA: it can travel long distances to the cell body, be packaged into EVs, and be secreted. Secretion of TrkA via EVs appears to be regulated by its own downstream effector cascades, raising intriguing future questions about novel functionalities associated with TrkA^+^ EVs. Functional and recipient studies in the future will discover the purpose of secreting EVs containing phosphorylated activated TrkA and the functional output of TrkA^+^ EVs in their intended recipient cells.

## Materials and methods

**Table:** 

Reagent or resource	Source	Identifier
Antibodies		
Rabbit anti-cytochrome C	Abcam	Cat # ab133504; RRID:AB_2802115
Rabbit anti-CD63	Abcam	Cat # ab217345; RRID:AB_2754982
Alexa Fluor 680 AffiniPure Donkey anti-Rabbit IgG	Jackson ImmunoResearch	Cat # 711-625-152 RRID: AB_2340627
Rabbit Anti- Calreticulin	Cell Signaling Technology	Cat # 12238S RRID: AB_2688013
Sheep Anti- Tyrosine Hydroxylase	Millipore	Cat # AB1542 RRID: AB_90755
Alexa Fluor 790 AffiniPure Donkey anti-Rabbit IgG	Jackson ImmunoResearch	Cat # 711-655-152 RRID: AB_2340628
Rabbit Anti- CD81	Cell Signaling Technology	Cat # 10037S RRID:AB_2714207
Rabbit Anti – Alix	Cell Signaling Technology	Cat # 92880 RRID:AB_2800192
Rabbit Anti-BLBP	Abcam	Cat # ab279649; RRID:AB
Chicken Anti-MAP2	EnCor Biotechnology	Cat # CPCA-MAP2 RRID: AB_2138173
Anti- FLAG DYKDDDDK tag (D6W5B) Rabbit mAb Alexa R 488	Cell Signaling Technology	Cat # 15008S Lot 2 and 3 RRID: N/A
Biological samples		
NGF	In house, purified from mouse salivary glands	
Chemicals, peptides, and recombinant proteins		
Poly-D-Lysine	Sigma	Cat # P7886
Prime XV IS-21	Sigma	Cat # 91142
Hyaluronidase	Sigma	Cat # H3884
Collagenase	Worthington	Cat # LS004196
Laminin	Invitrogen	Cat # 23017-01
BSA	Sigma	Cat # A9647
Polyacrylamide gels 4–12%	Genscript	Cat # M00654
Trypsin	Sigma	Cat # T4799
WGA-AF488	Fisher Scientific	Cat # W11261
PBS	Gibco	Cat # 14190-144
Milk	Lab Scientific	Cat # M0841
Beta mercaptoethanol	BioRad	Cat # 161-0716
DMEM no phenol	Gibco	Cat # 31053-028
GlutaMAX	Gibco	Cat # 35050-061
FBS	R & D Systems	Cat # S11195H
LY294002	Sigma	Cat # L9908
U-73122	Sigma	Cat # U6756
DAPI	ThermoFisher	Cat # D3571
Experimental models: organisms/strains		
C57 Bl/6 J mice	Jackson Laboratory	
TrkA^FLAG/FLAG^	Gift from D. Ginty	Harvard
Hardware, software and algorithms		
Odyssey CLx	LI-COR	
Optima TLX Ultracentrifuge	Beckman-Coulter	
Trans-Blot Turbo Transfer	Bio Rad	
Image J		https://imagej.nih.gov/ij/
Tecnai F20 Twin Electron Microscope	FEI	
Prism 9	Graphpad	graphpad.com
ZetaView PMX-120	Particle-Metrix	particle-metrix.com
Illustrator	Adobe	adobe.com
Other		
Microcentrifuge tubes	USAscientific	Cat # 1415-2500
Tissue culture plates	Fisher Scientific	Cat # 150628
Polycarbonate centrifuge tubes	Beckman	Cat # 343778
Sylgard 184 Silicone elastomer kit	Krayden	Cat # DC2065622

### Animals

All animal use complied with the Association for Assessment and Accreditation of Laboratory Animals Care policies and was approved by the University of Virginia Animal Care and Use Committee protocol #3422 (Winckler lab) and protocol #3795 (Deppmann lab). All mice were maintained on a C57Bl/6 J background. Males and females were mixed in all experiments. Mouse lines used: C57BL/6 J and TrkA^FLAG/FLAG^ ^15^. All methods were performed in accordance with ARRIVE (Animal Research: Reporting of In Vivo Experiments) guidelines (https://arriveguidelines.org) and regulations.

#### ARRIVE guidelines

All cells used in this study were derived from postnatal day 3 mice (*Mus Musculus*). Cells from both male and female mice were pooled. All mice were maintained on a C57Bl/6 J background. Specific strains used are mentioned in the appropriate results and figure legends. Each experiment was repeated at least three times on three independent litters of mice (6–10 pups per litter) and appropriate controls were included. The total number of animals used in this study was 148 mouse neonates. Dissected tissue from mouse neonates was pooled before trituration and plating of cells, therefore all cells were randomized between control and treatment groups. No animals were excluded from analysis. Outcome measures, experimental procedures, and statistical methods are described below. Results with appropriate descriptive statistics are described in the figure legend for each experiment.

### Primary sympathetic neuronal cultures

Postnatal day 3 (P3) mouse pups were euthanized by decapitation and the superior cervical ganglia were microdissected as previously described, and kept in ice-cold DMEM until enzymatic digestion^16^. Ganglia were transferred to an enzymatic solution containing 0.01 g/mL BSA, 0.4 mg/mL hyaluronidase, and 4 mg/mL collagenase for 20 min at 37 °C. This solution was aspirated off and replaced with a 2.5% trypsin solution for 15 min at 37 °C. Cells were then washed in DMEM containing 10% FBS 3 × and then subjected to trituration using a P1000 pipette and then a P200 pipette. Cells were then spun down at 300 × g and resuspended in complete media. A small 10 µL aliquot of cells was counted on a hemocytometer. Cells were plated at a density of no less than 100,000 cells in a 12-well plate that had been precoated with poly-D-lysine and 1 mg/mL laminin and washed 3 × with sterile PBS. Cells were kept in an incubator at 37 °C at 10% CO_2_ and the media was changed every 48 h. Because serum is known to contain EVs, cells were grown in serum-free complete media supplemented by Prime XV IS-21. Complete media contains the following: DMEM without phenol red, GlutaMAX, Prime XV IS-21, and 50 ng/mL NGF.

### Compartmentalized WGA feeding assay

Sympathetic neurons were dissected as described above and dissociated neurons were plated in microfluidic devices as previously described^36,37^. Cells were plated at a density of 14,000 cells per MFD. To encourage axonal crossing of the microgrooves, neurons were exposed to 30 ng/mL NGF in the CB chamber and 80 ng/mL NGF in the DA chamber. At 6 DIV, 150 µL of complete media was added to the CB chamber and 100 µL of WGA-AF488 (1:200) in complete media was added to the DA chamber. Conditioned media was collected from the CB chamber 15 h after the addition of WGA-AF488 and EVs were isolated.

### Compartmentalized Anti-FLAG antibody feeding assay

Sympathetic neurons from *TrkA*^*FLAG/FLAG*^ animals were dissected and plated at 14,000 cells per microfluidic device as previously described^36,37^. At 6 DIV, 150 μL of complete media was added to the CB chamber and 100 μL of anti-FLAG-AF488 antibody (1:200) in complete media was added to the DA chamber. Conditioned media was collected from the CB chamber 15 h after the addition of the anti-FLAG-AF488 antibody and EVs were isolated. For inhibitor treatments, 100 μL of complete media containing inhibitors (LY294002 50 μM, U-73122 1 μM, or 0.1% v/v DMSO) was added to the CB chamber and 150 μL of anti-FLAG-AF488 antibody (1:200) in complete media was added the DA chamber. Conditioned media was collected from the CB chamber 15 h after the addition of the anti-FLAG-AF488 and inhibitors and EVs were isolated.

### EV isolation and characterization

Methods of EV isolation and characterization were performed in accordance with the guidelines set forth by the International Society for Extracellular Vesicles’ position paper entitled “Minimal Information for the Study of Extracellular Vesicles”^12^.

#### EV isolation and differential centrifugation

Conditioned media was collected from cells after 48 h and placed into 1.5 mL microcentrifuge tubes on ice. In experiments using mass cultures, conditioned media from a single well in a 12-well plate was used. In experiments using MFDs, conditioned media collected from the CB chamber of six MFDs were combined. The conditioned media was then centrifuged at 300×*g* for 10 min at 4 °C to pellet the cells. The supernatant was transferred to a clean 1.5 mL microcentrifuge tube and centrifuged at 2,000×*g* for 10 min at 4 °C to pellet dead cells. The supernatant was transferred to a clean 1.5 mL microcentrifuge tube and spun at 20,000×*g* for 30 min. The pellet from this step is the P20 fraction. The supernatant was transferred to polycarbonate tubes subjected to ultracentrifugation at 100,000×*g*_max_ (rotor: TLA 120.2; k -factor: 42; 53,000 rpm) for 70 min at 4 °C. The pellet from this step is the P100 fraction.

#### Nanoparticle tracking analysis

NTA was conducted using the ZetaView PMX 120 equipped with a 488 nm laser and a long wave pass filter (cutoff 500 nm) and CMOS camera. Samples were diluted to 1 mL in PBS prior to analysis. Each sample was measured at 11 different positions over 3 cycles ensuring a minimum number of 1000 traces were recorded. Two technical replicates were performed for each sample. Samples were recorded at 25 °C, pH 7.0 with a shutter speed and camera sensitivity of 75 at 30 frames per second. Automatically generated reports of particle counts were checked and any outliers were removed to calculate the final concentration.

#### Western blot

All samples were lysed directly in 1.2X Laemmli sample buffer containing 5% BME and boiled for 5 min. Laemmli sample buffer recipe: 4% SDS (10% (w/v), 20% glycerol, 120 mM 1 M Tris–Cl (pH 6.8), and 0.02% (w/v) bromophenol blue in water. Sympathetic cultures were washed with PBS and lysed directly on the plate with 200 μL of 1.2X Laemmli sample buffer. P20 and P100 fractions were lysed directly in micro/ultracentrifuge tubes with 30 μL of 1.2X Laemmli. The sample buffer was pipetted up and down 50 times along the walls of the tubes to collect the entire pellet. Samples were run on 4–12% polyacrylamide gels with 7 μL of cell pellet fractions and 15 μL of P20 and P100 fractions loaded per well. Protein gels were transferred to nitrocellulose membranes using the Trans-blot turbo, blocked in 5% milk for 1 h, and incubated in primary antibody (Alix 1:1000, CD63 1:1000, CD81 1:1000, Cytochrome C 1:5000, Calreticulin 1:4000) diluted in 5% milk 0.1% TBST overnight at 4 °C on a rocker. Membranes were then washed 3 × with 0.1% TBST and secondary antibodies (1:20,000) diluted in 0.1% TBST were incubated for 1 h at room temperature. Blots were imaged using the Odyssey CLx imager and exposure was determined automatically by the software.

#### Electron cryo-microscopy

Cryo-TEM was performed by the molecular electron microscopy core at UVA. P20 and P100 fractions were resuspended in 30 mL PBS. An aliquot of the sample (~ 3.5 μL) was applied to a glow-discharged, perforated carbon-coated grid (2/1-3C C-Flat; Protochips, Raleigh, NC), manually blotted with filter paper, and rapidly plunged into liquid ethane. The grids were stored in liquid nitrogen, then transferred to a Gatan 626 cryo-specimen holder (Gatan, Warrrendale, PA) and maintained at ~ 180 °C. Low-dose images were collected on a Tecnai F20 Twin transmission electron microscope (FEI {now ThermoFisher Scientific}, Hillsboro, OR) operating at 120 kV. The digital micrographs were recorded on a TVIPS XF416 camera (Teitz, Germany).

### Immunocytochemistry

Cells were fixed in 4% PFA for 20 min at room temperature in the MFDs. Cells were washed 3 times with 1X PBS and then blocked and permeabilized in 5% normal donkey serum and 0.2% TritonX-100 for 20 min. Primary antibodies were diluted in 1% BSA and applied overnight at 4 °C. Secondary antibodies were diluted in 1% BSA and added for 30 min at room temperature. MFDs were washed 3 × with 1 × PBS and imaged on an inverted Zeiss 980 microscope with an Airyscan detector using a 40X oil objective (NA 1.3).

### Statistics and measurements

Vesicles from micrographs were measured at their widest diameter using the segment tool in Image J. Cell counts were determined using the cell counter plugin in Image J. Cell type was determined using the following: all cells (DAPI^+^), SCG neurons (MAP2^+^; FLAG^+^ double positive) and satellite glia (BLBP^+^). Cells were classified as “other” if they were DAPI^+^, but did not show staining against MAP2, FLAG, or BLBP. Soma and nuclei area was determined by drawing an ROI around the cell body of SCG neurons (MAP2^+^;FLAG^+^ double positive) or nucleus (DAPI^+^) and measuring the area in Image J. Statistical analyses were performed using Prism 9 software. All values are shown as mean ± SEM. Differences between samples were determined using unpaired, two-tailed t-tests. Statistical significance is a p-value < 0.05. The p-values are denoted on top of each bracket pair.

## Supplementary Information

Supplementary Information.

## Data Availability

Datasets generated and analyzed in this current study are available from the corresponding authors upon reasonable request.
