# Supplementary material for: Sympathetic neurons secrete retrogradely transported TrkA on extracellular vesicles
Source: Sci Rep. 2023 Mar 4;13:3657. doi: 10.1038/s41598-023-30728-3 (PMC9985603; doi:10.1038/s41598-023-30728-3)
Supplement: Supplementary file 1 — Supplementary Information. [file 41598_2023_30728_MOESM1_ESM.pdf]

Supplementary Information for “Sympathetic neurons secrete retrogradely transported TrkA on extracellular vesicles”

Authors:
Ashley J Mason, Austin B Keeler, Farah Kabir, Bettina Winckler, Christopher Deppmann

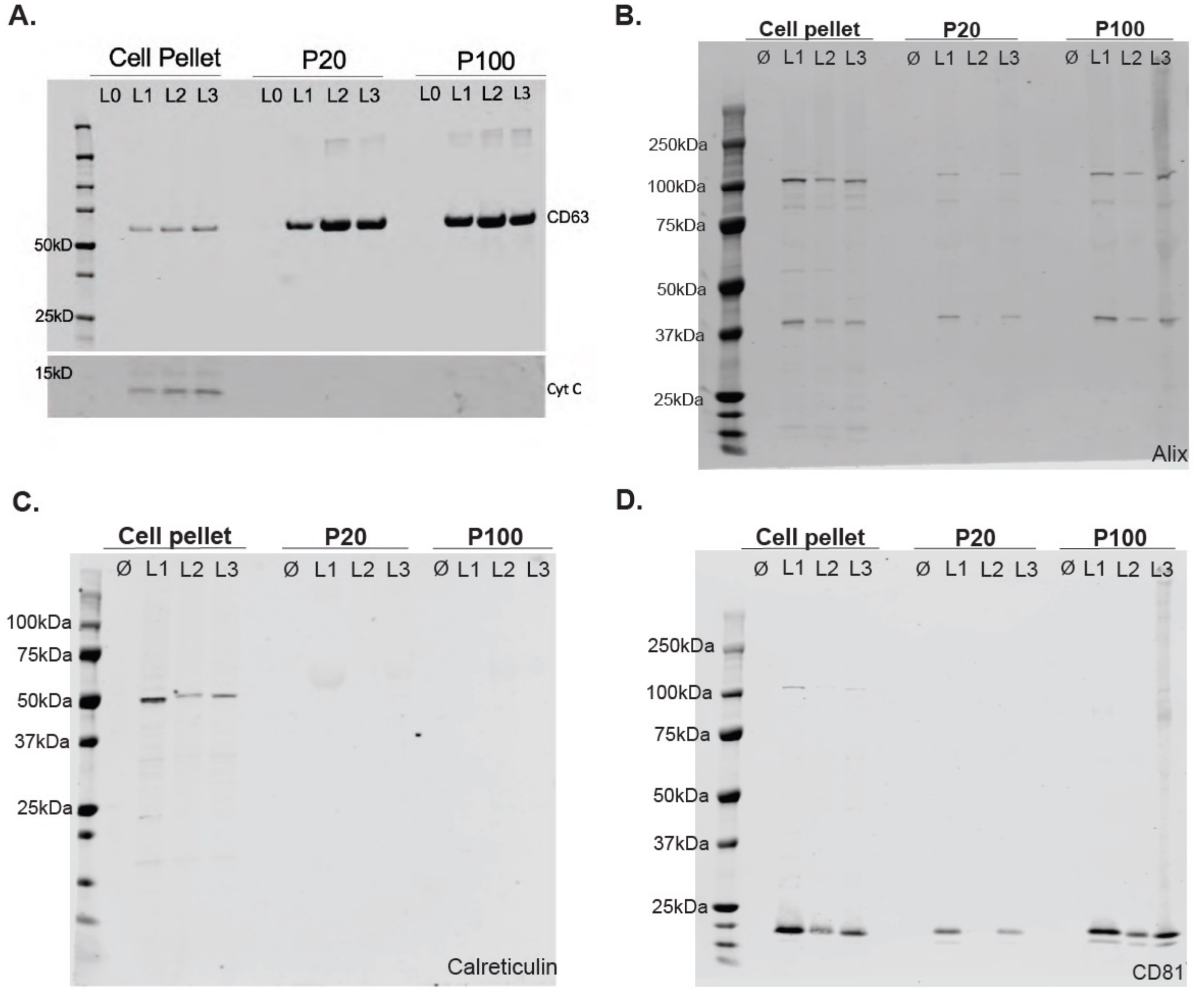

Supplementary Figure S1. Full-length immunoblots of EV and cell lysate markers that are shown in Figure 1D.

A. Full immunoblot that was physically cut in half before the addition of primary antibody against the EV marker tetraspanin, CD63 and the mitochondrial marker, Cytochrome C (Cyt C). **B.** Full immunoblot of the EV marker and accessory ESCRT protein, Alix. Full-length Alix is predicted to run around 95 kDa. The smaller band is either non-specific or represents a breakdown product of full-length Alix. **C.** Full immunoblot of the endoplasmic reticulum marker, Calreticulin **D.** Full immunoblot of the EV marker tetraspanin, CD81 from the same blot in panel (**B)** that was stripped and re-probed.

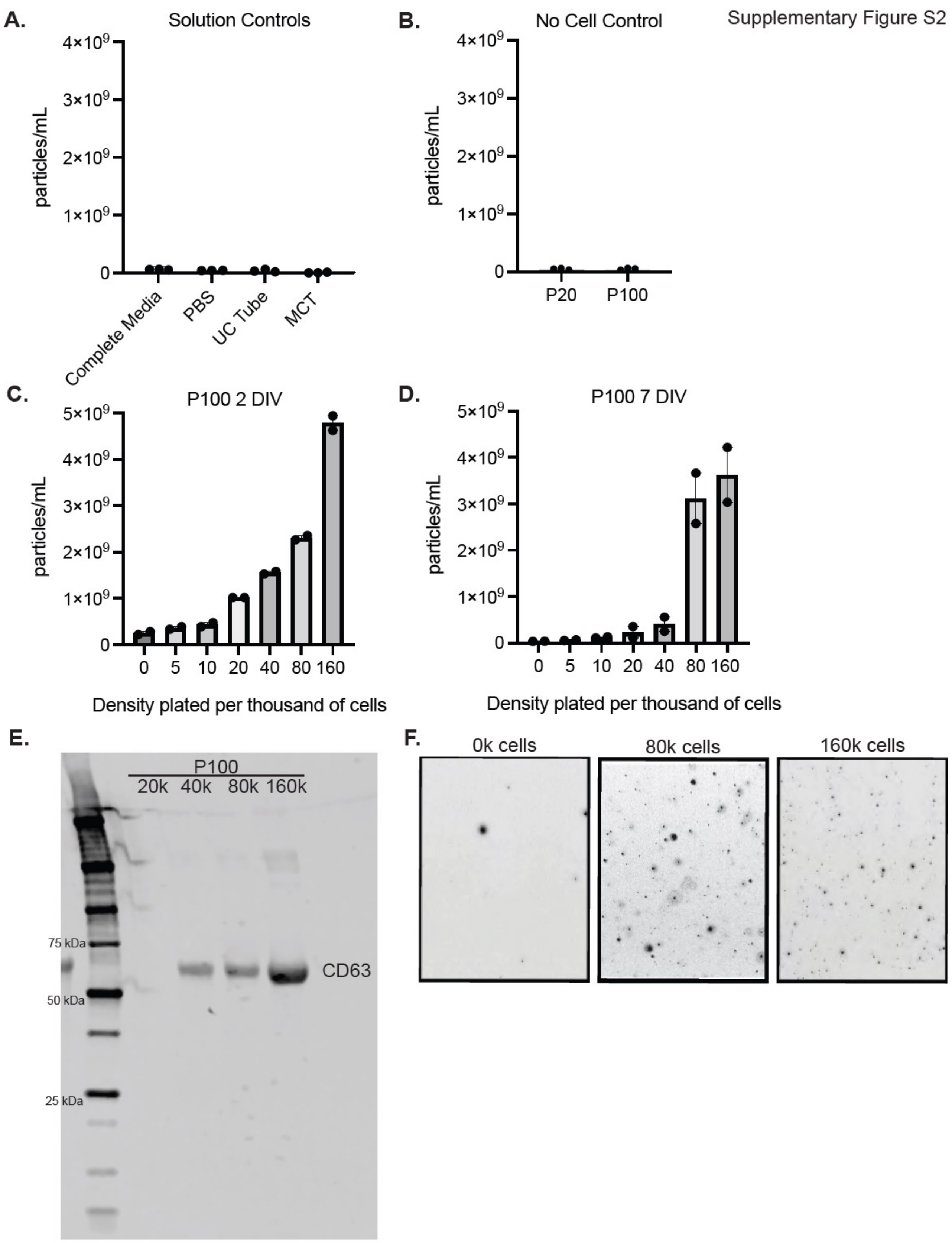

Supplementary Figure S2

Supplementary Figure S2. Density and days in vitro affect EV production

A. One milliliter of each undiluted solution condition was analyzed by NTA for non-EV scattering particles. Complete media, PBS, UC tube (PBS that sat in a polycarbonate centrifuge tube for 3 hours), MCT (PBS that sat in a microcentrifuge tube for three hours). **B. "**No cell" only control consisting of complete media that was plated in a 12-well plate and changed every 48 hours before collection and differential centrifugation. Shown is mean ± SEM for three replicates. **C-D.** Density and DIV curve from P100 fraction. Cells were plated at the density shown on the x-axis and grown for either 2 DIV (**C)** or 7 DIV **(D)** before CM was collected for EV isolation and NTA analysis. Shown is mean ± SEM for two replicates. **E.** Immunoblot analysis of CD63 at different densities of plated SCG cells. **F.** Still frames captured from NTA videos at t=30 secs of EVs from different plated cell densities.

Supplementary Figure S3

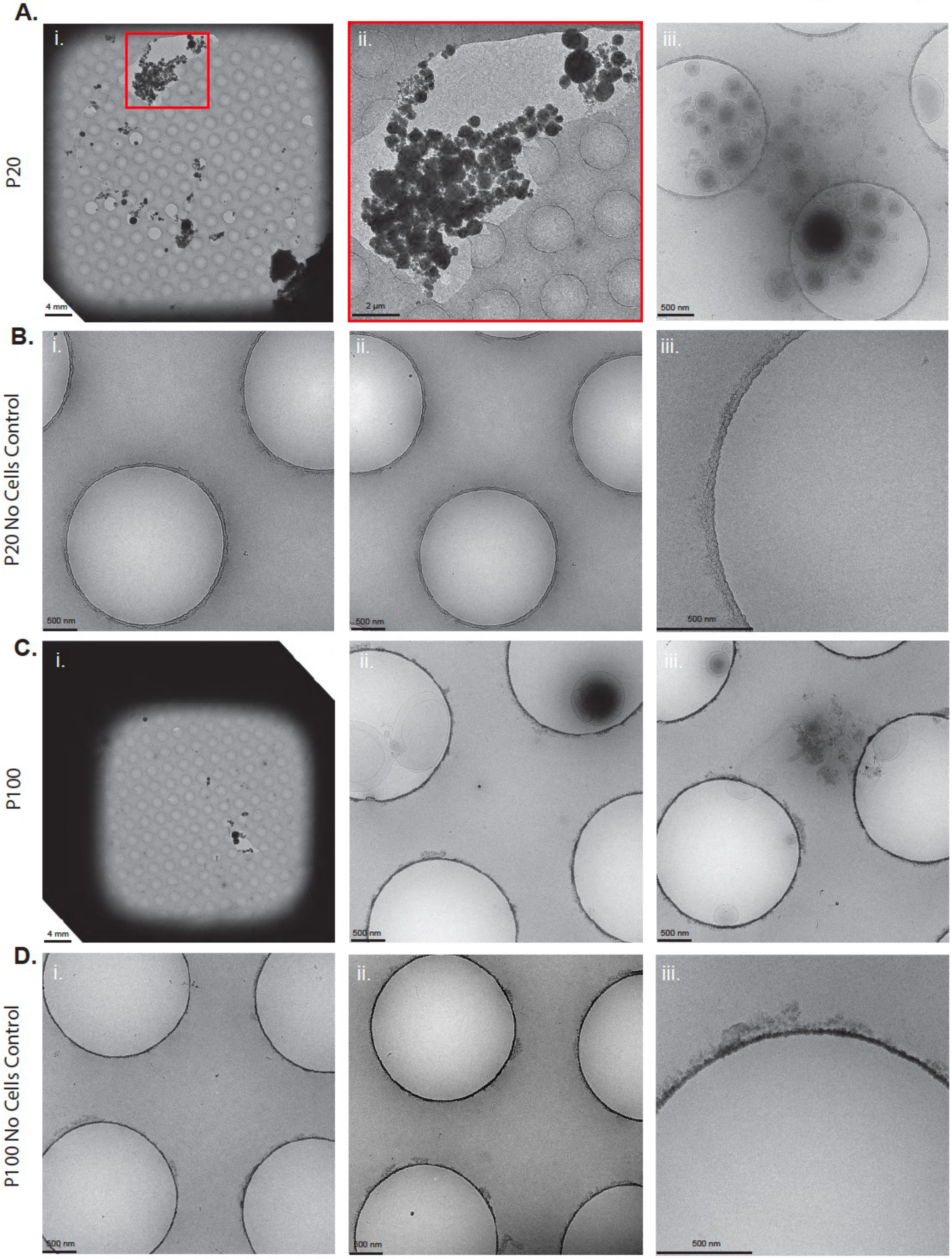

Supplementary Figure S3. Low magnification cryo-EM micrographs of EVs

A. Low magnification micrographs of the P20 fraction. i. Shown are large aggregates which cannot be measured as discrete EVs. Scale bar is 4 mm. ii. Zoomed in view of the red boxed inset in i. Scale bar is 2 µm. iii. Discrete double membrane-enclosed EVs are discernable with different-sized EVs with different electron densities. Scale bar is 500 nm. **B.** Low magnification micrographs of the P20 “no cell” controls (i., ii., and iii). Scale bar is 500 nm for all. **C.** Low magnification micrographs of P100 fraction. i. Full grid view of the P100 fraction with noticeably fewer large aggregates as compared to the P20 fraction. Scale bar is 4 mm. ii. EVs with interesting shapes and electron densities are viewable in the perforations. Scale bar is 500 nm. iii. Cluster of heterogeneous EVs. Scale bar is 500 nm. **D.** Low magnification micrographs of P100 “no cell” controls (i., ii., iii.). Scale bar is 500 nm for all.

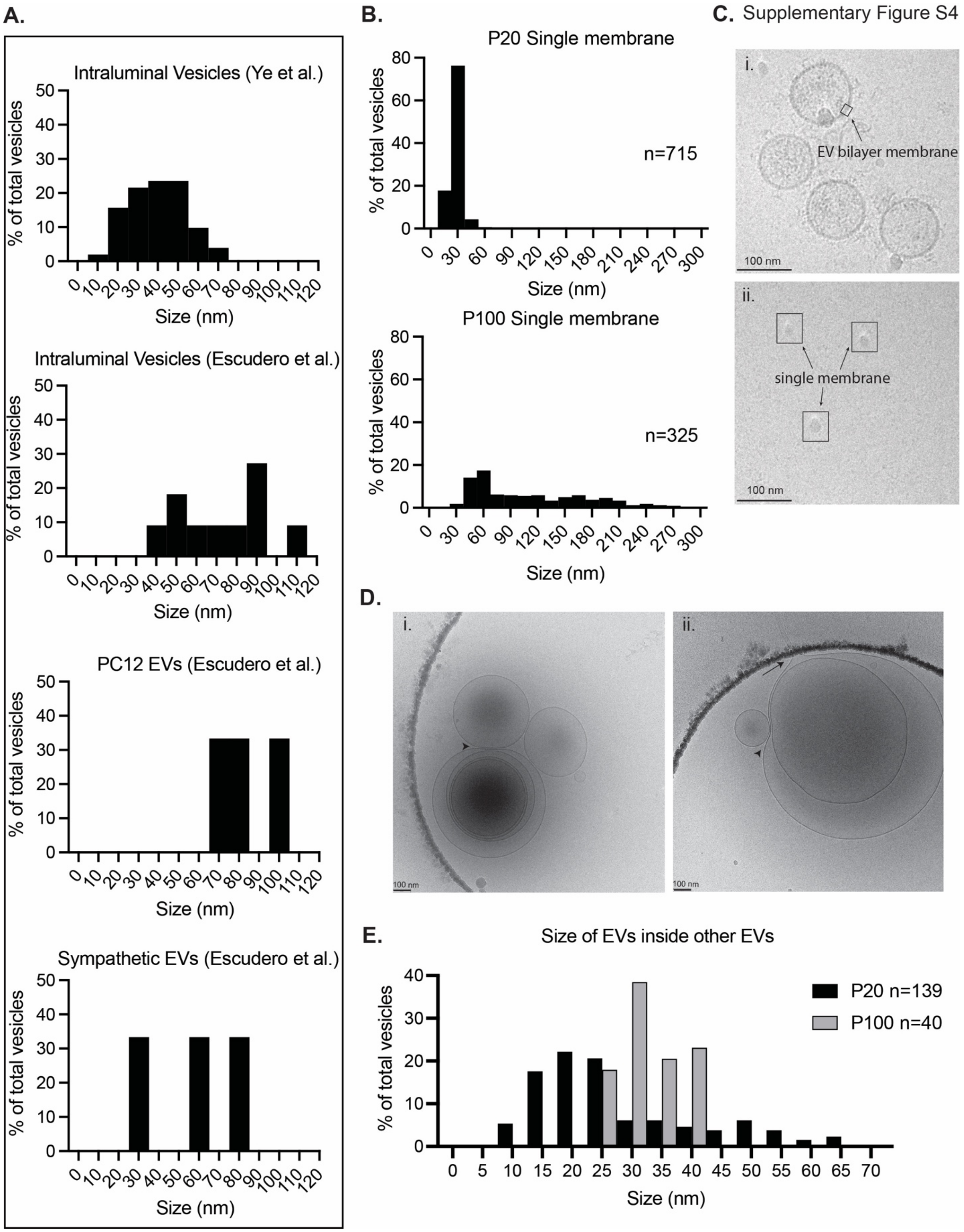
Supplementary Figure S4
A.
Intraluminal Vesicles (Ye et al.)
% of total vesicles
Size (nm)
Intraluminal Vesicles (Escudero et al.)
% of total vesicles
Size (nm)
PC12 EVs (Escudero et al.)
% of total vesicles
Size (nm)
Sympathetic EVs (Escudero et al.)
% of total vesicles
Size (nm)
B.
P20 Single membrane
% of total vesicles
n=715
Size (nm)
P100 Single membrane
% of total vesicles
n=325
Size (nm)
C.
i.
EV bilayer membrane
100 nm
ii.
single membrane
100 nm
D.
i.
100 nm
ii.
100 nm
E.
Size of EVs inside other EVs
% of total vesicles
P20 n=139
P100 n=40
Size (nm)

Supplementary Figure S4. Heterogeneity in size and morphology of sympathetic EVs

A. Size distribution histogram showing intraluminal vesicles (ILVs) from sympathetic neuron transmission electron micrographs from Ye et al., 2018[5] (mean diameter 52.8 nm) and Escudero et al., 2014[14] (mean diameter 79.9 nm) as well as EVs derived from NGF-differentiated PC12 cells (mean diameter 80.0 nm) and sympathetic EVs (mean diameter 58.3 nm) from Escudero et al., 2014[14]. **B.** Size distribution histogram of single membrane enclosed EVs from the P20 fraction (top) (n= 715 EVs, mean ± SEM is 21.04 nm ± 15.59 nm) and P100 fraction (bottom) (n= 325 EVs, mean ± SEM is 159.03 nm ± 191.56 nm) for three biological replicates. **C.** Zoomed in micrograph of small EVs. i. Small EVs with a distinct double membrane lipid bilayer. Scale bar is 100 nm. ii. Sub 30 nm diameter exomeres with only a single membrane. Scale bar is 100 nm. **D.** Heterogeneity in size and structure of EVs. i. micrograph demonstrating EVs inside EVs (data quantified in E), electron dense EVs, and EVs deforming around each other (arrowhead). ii. Micrograph showing EVs inside EVs, EV membranes deforming around each other (arrowhead), and long tubule-like projections from EV membranes (arrow). Scale bar is 100 nm for all images. **E.** Size distribution histogram of EVs enclosed inside of other EVs for both the P20 (mean ± SEM is 35.5 nm ± 26.79 nm) and P100 fraction (mean ± SEM is 24.59 nm ± 5.44 nm).

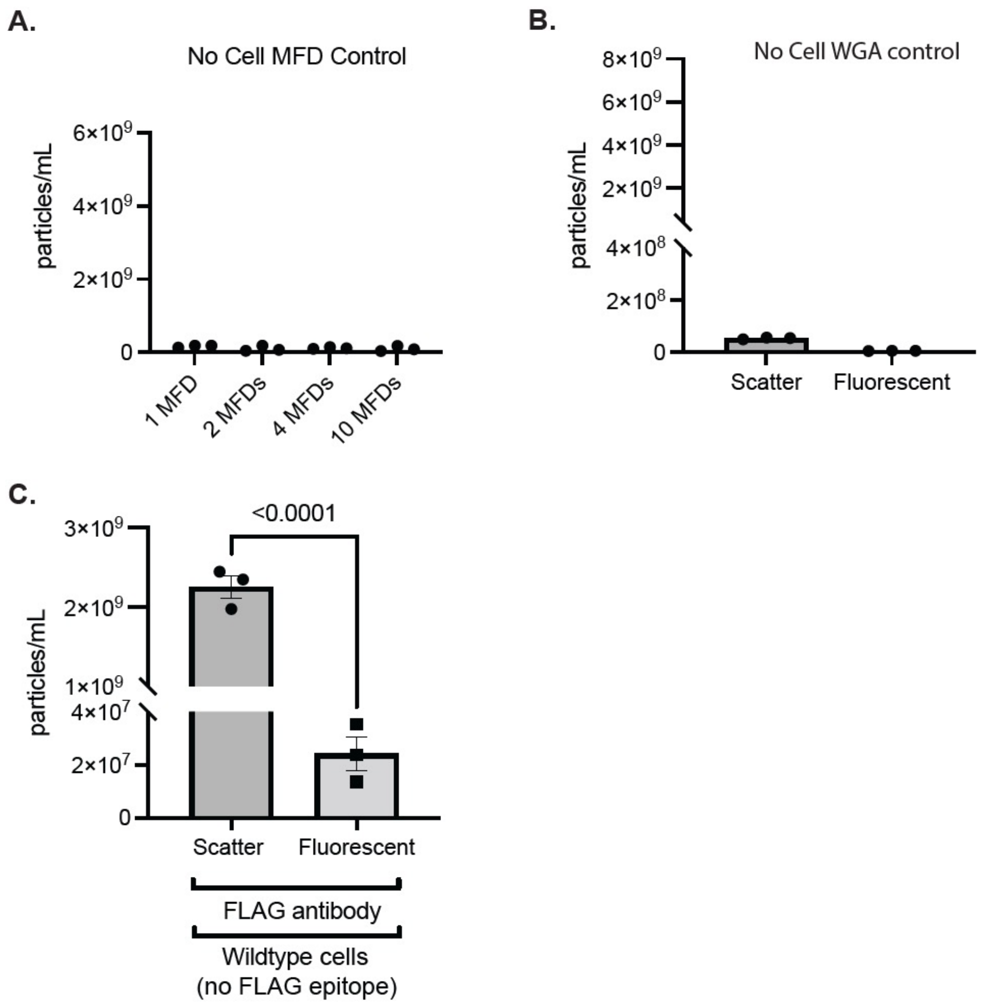

Supplementary Figure S5. Control for compartmentalized FLAG feeding assays
A. "No cell" MFD control consists of complete media that was plated and pooled from either 1, 2, 4, or 10 MFDs and changed every 48 hours before collection and differential centrifugation. Particle counts were 1.28 x 10^7, 2.23 x 10^7, 4.86 x 10^7, and 8.32 x 10^7 particles/mL for 1, 2, 4, and 10 MFDs, respectively. **B.** Quantification of the total number of particles (scatter) and the number of fluorescent (WGA-AF488$^+$) particles collected from the P100 fraction after WGA-AF488 addition to the DA chamber of MFDs containing no cells. **C.** Quantification of the total number of particles (scatter) and the number of fluorescent (Anti-FLAG-AF488$^+$) particles collected from the P100 fraction after Anti-FLAG-AF488 addition to the DA chamber of MFDs containing wildtype (no FLAG epitope) SCG neurons. **A-C.** Shown is the mean ± SEM for three biological replicates. P-values are indicated above the pairwise brackets.

Supplementary Figure S6

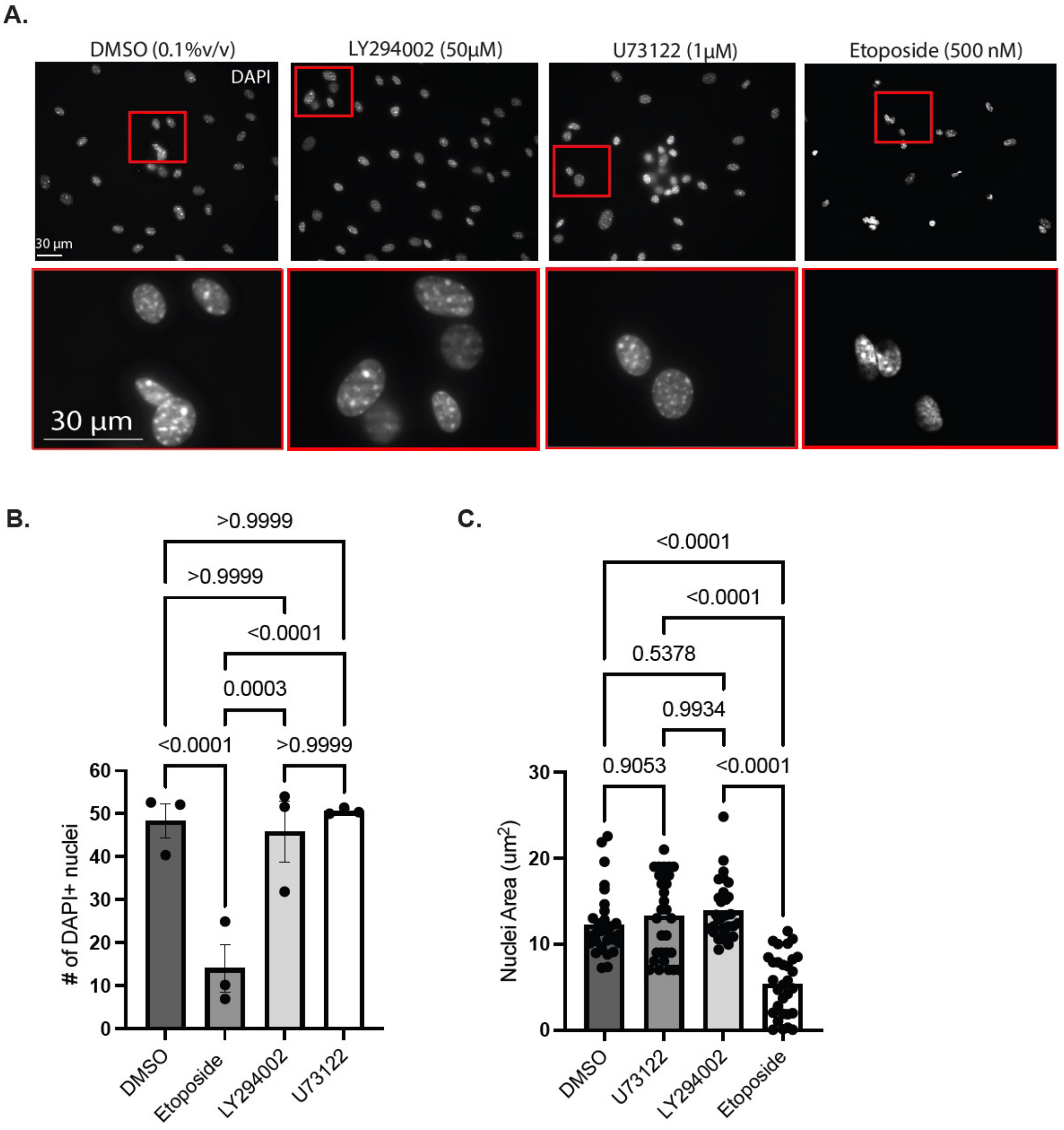

Supplementary Figure S6. TrkA inhibitors do not affect sympathetic culture viability

A. Images of nuclei from compartmentalized sympathetic cultures treated for 15 hours with DMSO (inhibitor solvent), etoposide (positive control to promote cell death), or inhibitors in the cell body chamber. Zoomed in view of the red boxed inset is below. Scale bar is 30 μm. **B.** Quantification of the number of total cells (DAPI$^+$) present in each condition. Shown is the mean ± SEM for three biological replicates from 10 fields of view per replicate. **C.** Quantification of the nucleus area from 30 cells in each condition. **B-C.** P-values are indicated above the pairwise brackets.

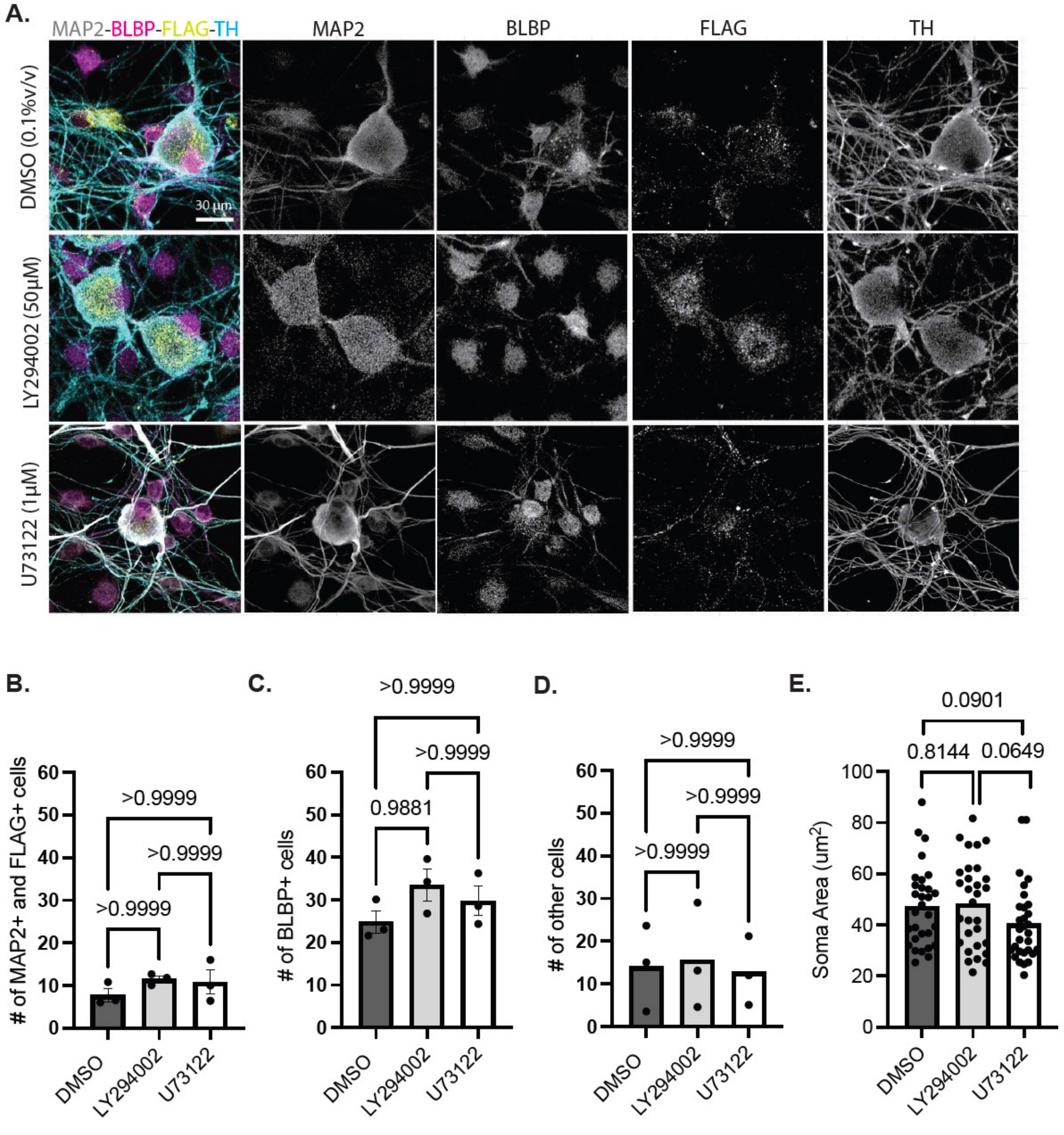

Supplementary Figure S7. TrkA inhibitors do not affect SCG neuron morphology
A. Images of the cell bodies of compartmentalized SCG neurons treated with DMSO (control) or inhibitors in the cell body chamber and anti-FLAG-AF488 antibody in the distal axon chamber for 15 hours. Staining against MAP2 (somatodendritic domain), BLBP (satellite glia), anti-FLAG-AF488, and TH (SCG neuron). Scale bar is 30 μm. **B-D.** Quantification of the number of SCG neurons ($MAP2^+$; $FLAG^+$ double positive), satellite glia ($BLBP^+$), and other cells ($DAPI^+$ and $BLBP^-$; $FLAG^-$; $MAP2^-$) from each condition. Shown is mean ± SEM for three biological replicates from 10 fields of view per replicate. **E.** Quantification of the somata area of 30 SCG neurons that were $MAP2^+$; $FLAG^+$ double positive. P-values are indicated above the pairwise brackets.
